# Shear Stress-Dependent Targeting Efficiency Using Self-Assembled Gelatin–Oleic Nanoparticles in a Biomimetic Microfluidic System

**DOI:** 10.3390/pharmaceutics12060555

**Published:** 2020-06-16

**Authors:** Taehee Kang, Chulhun Park, Nileshkumar Meghani, Thao T.D. Tran, Phuong H.L. Tran, Beom-Jin Lee

**Affiliations:** 1 College of Pharmacy, Ajou University, Suwon 16499, Korea; kangthh@gmail.com (T.K.); chulhun@ualberta.ca (C.P.); meghani.nilesh@gmail.com (N.M.); 2 Faculty of Pharmacy & Pharmaceutical Sciences, University of Alberta, Edmonton, AB T6G 2E1, Canada; 3 Department for Management of Science and Technology Development, Ton Duc Thang University, Ho Chi Minh City, Vietnam; trantruongdinhthao@tdtu.edu.vn; 4 Faculty of Pharmacy, Ton Duc Thang University, Ho Chi Minh City, Vietnam; 5 School of Medicine, Deakin University, Geelong 3217, Australia; phuong.tran1@deakin.edu.au; 6 Institute of Pharmaceutical Science and Technology, Ajou University, Suwon 16499, Korea

**Keywords:** gelatin–oleic conjugate, self-assembly, fattigation-platform NPs, biomimetic shear stress, cell dynamic environment, cellular uptake, paclitaxel, coumarin-6, inhibitory concentration

## Abstract

Cellular properties and microenvironments, as well as the characteristics of nanoparticles (NPs), affect the cellular uptake and cytotoxic effects of drug-loaded NPs. Since there is fluid flow in the human blood system, fluid flow also affects the drug delivery efficiency of NPs. This study aimed to evaluate the cellular behaviors of drug-loaded soft NPs on A549 cancer cells under different levels of shear stress (0.5, 5, and 50 dynes/cm^2^) in the biomimetic microfluidic system. The soft self-assembled NPs were formed by the gelatin–oleic conjugate (GOC). The poorly water-soluble coumarin-6 or paclitaxel (PTX) were used as model markers for encapsulation within self-assembled NPs (C-GONs or PTX-GONs, respectively). The cellular uptake of C-GONs was found to be improved with shear-stress dependence. The inhibitory concentration (IC_50_) of PTX-GONs at 0.5, 5, and 50 dynes/cm^2^ was 0.106 µg/mL, 0.108 µg/mL, and 0.091 µg/mL, respectively, as compared to 0.138 µg/mL in a static condition. The cell killing efficiency of PTX-GONs was increased in the highest shear stress of 50 dynes/cm^2^ in the static condition, and other levels of shear stress in dynamic conditions.

## 1. Introduction

Numerous studies have demonstrated the primary cellular uptake mechanism and interaction of nanoparticles (NPs) at the single-cell level [1]. The properties of NPs, such as size, surface charge, and structural rigidity, as well as cellular properties and microenvironments, can affect the cellular uptake and cytotoxic effects of anticancer drug-loaded NPs [2]. Understanding the interaction between NPs and cells is essential for designing drug delivery vehicles for cancer treatment [3]. However, the therapeutic results obtained from the clinical trials of the NPs-based drug delivery system often differ from the outstanding performances of cell-based experimental studies [4]. One of the reasons for this phenomenon is the use of static conditions during a cell-based experiment, which cannot satisfactorily mimic the dynamic in vivo environments [5]. Additionally, there are reasons why the outstanding performances of the NP system, e.g., by dysregulating the growth of cancers in animal studies, do not assure clinical success in the pharmaceutical market [6].

Interstitial flow is the movement of biologic fluid between the extracellular matrix and the systemic circulation system [7]. Blood or lymphatic flow encountering the vessel wall induces frictional forces expressed in the force divided by unit per area (dynes/cm^2^) [7]. Without any turbulence or convective flow in the straight regions of arteries, steady laminar flow patterns are mainly exhibited. Irregular blood flow patterns generate microfluidic shear stress in the stenotic arteries and blood vessels surrounding tumors [8]. In tumor cells, interstitial shear stress plays a vital role in promoting tumor metastasis, lymphatic drainage, and drug delivery efficiency to cancer cells [9]. Previous studies have proven that the dependency of cellular interaction was variable with the type of NP. For example, the drug delivery efficiency of poly(lactic-co-glycolic acid)-based NPs was decreased by high fluid shear force [10]. The improved drug delivery efficiency of liposome and polystyrene NPs occurred in higher shear stress conditions [11,12]. Other studies have confirmed that the surface charge of NPs can change cellular interaction in dynamic conditions, with positively charged NPs having a higher uptake into myoblast cells, whereas negatively charged NPs showed a lower uptake intensity [13]. These findings suggest that different features of NPs could significantly affect their cellular uptake in a biomimetic dynamic microenvironment. The levels of shear stress are highly variable in the tumor metastasis-related microenvironment. Different levels of microfluidic shear stress may affect the intracellular uptake of NPs during interactions between NPs and cells. Additionally, non-invasive cell-based experiments on new formulations that are newly developed through mimicking the human body systems, including artificial organs and organ-on-a-chip, are actively being researched [14,15]. However, the mechanistic understanding of drug delivery with fluidic shear stress remains mostly unexplored.

To investigate the effect of diverse biomimetic shear stress on NPs, fattigation-platform gelatin–oleic NPs (GONs) were prepared via the self-assembly of the amphiphilic gelatin–oleic conjugate (GOC) using a desolvation method, as previously established in our research group [16]. Among the different types of materials used to prepare fattigation-platform NPs, gelatin is a versatile biomaterial because it is non-toxic, biocompatible, and also easy to modify the biochemical structures [17]. Oleic acid (OA), which is generally recognized as a safe material, was conjugated to gelatin through the 1-ethyl-3-(3-dimethylamino propyl) carbodiimide/*N*-hydroxy succinimide (EDC/NHS) reaction, as a fattigation-platform technique, and the hydrophobic interaction among gelatin molecules was increased to form the amphiphilic GOC. The self-assembled GONs had proven performance in satisfying patients’ unmet needs for enhancing the solubilizing capacity and cancer-targeting effects of various model drugs [18,19]. Notably, the anti-cancer activity and of PTX are mainly hindered by their poor solubility [20]. As defeating the challenges of poorly water-soluble PTX, the formation of highly nanonized drugs in self-assembled domains should consider the critical molecular mechanism with inducing the absorption with freely solvated partitioning drug into micelles [21]. The fattigation-platform technique can achieve the scopes of solubilizing the poorly water-soluble drugs, having increased the solubility, widened the therapeutic window, and improved patient-centricity [22,23].

This study aimed to investigate the cellular uptake and cytotoxicity of fattigation-platform GONs on A549 cancer cells, according to the static and dynamic cell culture conditions. The dynamic cell condition was designed to mimic the extracellular environment through the established biomimetic microfluidic system (BMS). The physicochemical properties of drug-loaded GONs were evaluated by particle characterization, drug-loading capacity, and electron microscopes. The poorly water-soluble coumarin-6 and paclitaxel (PTX) were chosen as a fluorescent marker and a model drug, respectively. The coumarin-6-loaded GONs (C-GONs) were used for monitoring cellular behaviors via confocal microscopy and flow cytometry. The inhibitory concentration of paclitaxel-loaded GONs (PTX-GONs) was determined by a cell viability assay with different cell culture conditions (static or dynamic conditions; 0.5, 5.0, and 50.0 dynes/cm^2^).

## 2. Materials and Methods

### 2.1. Materials

Gelatin and OA were respectively obtained from the Kanto Chemical Co., Inc. (Tokyo, Japan) and Shinyo Pure Chemicals Co., Ltd. (Osaka, Japan). Glutaraldehyde solution and 1-Ethyl-3-(3-dimethylamino-propyl) carbodiimide and N-hydroxysuccinimide were purchased from Sigma Chemical Co. Ltd. (St. Louis, MO, USA). The 2-mercaptoethanol (2-ME) was supplied from Yakuri Pure Chemicals Co., Ltd. (Kyoto, Japan). HPLC grade solvents were supplied by Thermo Fisher (Waltham, MA, USA). All other chemicals of analytical grade were used without a purification process. Biomimetic microfluidic system components and a microfluidic cell chamber (µ-Slide VI 0.4) were purchased from Ibidi GmbH (Munich, Germany). Paclitaxel (PTX) was obtained from Daewoong Pharmaceutical Co. Ltd. (Seoul, Korea).

### 2.2. Preparation and Characterization of GONs, C-GONs, and PTX-GONs

#### 2.2.1. Synthesis of GOC

The GOC was synthesized by a slight modification of the protocols from our research groups [16]. Briefly, a dispersion of 150 µL of OA in 30 mL of 60% ethanol was dissolved by adding 37.5 μL of 1M NaOH. Then, 364.45 mg of EDC and 291.73 mg of NHS were dissolved into this solution. The resulting solution was shaken in an incubator (BioFree, Seoul, Korea) to activate the oleic acid solution at 37 °C at a shaking speed of 100 rpm for 20 min. One hundred and thirty-five microliters of 2-ME were added to inactivate the unreacted EDC and then shaken for 10 min under the same conditions.

Next, 200 mg of gelatin was completely dissolved in 8 mL of 60% ethanol solution. Two hundred and fifty microliters of NaOH (1 M) were added to the gelatin solution. Then, the gelatin solution was added to the activated oleic acid solution. The reaction of conjugation was carried out in a shaking incubator for 12 h at 37 °C at a shaking speed of 100 rpm. Finally, acetone as a precipitating solvent was poured into the gelatin–oleic acid conjugate (GOC) solution and the reacted suspension was then centrifuged at 4000 rpm, and the supernatant was discarded. After the repeated centrifugation process, the final GOC was washed twice with ethanol and distilled water to remove the unreacted agents. The final GOC was stored in a drying oven at 40 °C.

#### 2.2.2. Preparation of GONs Using a Desolvation Method

**Figure 1:**
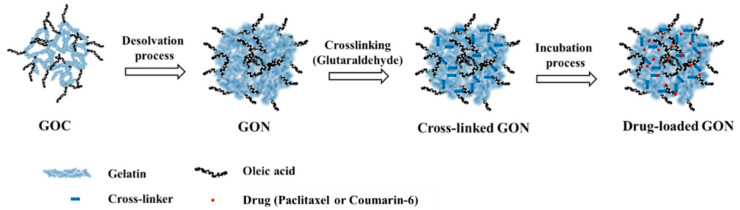
Schematic illustration of fattigation-platform Gelatin–Oleic Nanoparticles (GON) preparation and drug loading process.

The schematic illustration for the preparation and drug-loading process of fattigation-platform GONs was described in Figure 1. Firstly, 10 mg of GOC was dissolved in 3 mL of 50% ethanol. Four milliliters of anhydrous ethanol were gradually added to the GOC solution using a peristaltic pump and stirred at 700 rpm to convert the GOC solution into a colloidal state. After adding the 25 µL of 8% glutaraldehyde, the GOC solution was reacted for 10 h at 37 °C. Then, the cross-linked GONs were centrifuged at 12,000× *g* for 30 min. The GONs were washed three times with distilled water by centrifugation at 12,000× *g* for purification. Finally, purified GOCs were lyophilized at −50 °C for 2 days (Freeze Dryer, Ilshin Lab Co., Ltd., Gyeonggi-do, Korea).

#### 2.2.3. Preparation of C-GONs and PTX-GONs

Coumarin-6 and PTX were separately encapsulated within GONs by an incubation method. In this method, 10 mg of GONs were dispersed in water, and 10 μg of coumarin-6 or 1 mg of PTX were added at a shaking speed of 100 rpm for 24 h at 37 °C. The resulting solution was centrifuged, and the drug-loaded GONs (C-GONs, PTX-GONs) were purified and obtained.

### 2.3. Physical Characterization of GONs, C-GONs, and PTX-GONs

#### 2.3.1. Particle Size and Surface Charge Measurements of the NPs

The particle size and surface charge of the NPs (GONs, C-GONs, and PTX-GONs) were determined using a PAR-III Laser Particle Analyzer System (Otsuka Electronics, Osaka, Japan) with a He-Ne laser light source (5 mW) at a 90° angle. This measurement, called dynamic light scattering (DLS), established a non-invasive technique for determining the particle size and zeta-potential of NPs.

#### 2.3.2. Electron Microscopy of the NPs

The structure of the NPs was observed by transmission electron microscopy (TEM). Ten microliters of the NP solution were dropped on a copper grid covered with formvar-coated film and dried for 1 day before measurement by the TEM apparatus (TECNAI G2 F30 S-TWIN, FEI Company, Hillsboro, OR, USA). The surface morphologies of the NPs were visualized by field-emission scanning electron microscopy (FE-SEM). The solution samples of the NP formulation were dropped on the mounted carbon film. Then, they were coated with Au-Pd and determined by FE-SEM measurements (JSM 6700F, JEOL, Akishima, Japan).

#### 2.3.3. Drug Loading Content (DC) and Encapsulation Efficiency (EE)

In the drug loading process of the NPs, the unencapsulated PTX was collected from washed supernatants after centrifugation. Then, samples were analyzed using a UV/VIS spectrophotometer (DU730, Beckman Coulter, CA, USA) at 227 nm to detect the maximum wavelength of PTX. Finally, the DC and EE of drug-loaded GONs were calculated by the following equations by dividing the amount of encapsulated drug from the amount of drug initially added.

(1) $$\mathrm{DC}\;\left(\%\right)=\frac{\mathrm{Weight}\;\mathrm{of}\;\mathrm{the}\;\mathrm{drug}\;\mathrm{in}\;\mathrm{NPs}}{\mathrm{weight}\;\mathrm{of}\;\mathrm{NPs}}\times 100$$

(2) $$\mathrm{EE}\;\left(\%\right)=\frac{\mathrm{Amounts}\;\mathrm{of}\;\mathrm{the}\;\mathrm{encapsulated}\;\mathrm{drug}\;\mathrm{in}\;\mathrm{NPs}}{\mathrm{Amounts}\;\mathrm{of}\;\mathrm{the}\;\mathrm{initially}\;\mathrm{added}\;\mathrm{drug}}\times 100$$

### 2.4. Cell Culture in a Microfluidic Chamber

The human lung adenocarcinoma cell line A549 (KCLB No. 10185) was obtained from the Korean Cell Line Bank (Seoul, Korea). The cell culture RPMI 1640, supplemented with fetal bovine serum (10%) and penicillin/streptomycin (1%), was used for culturing the A549 cancer cell line. Thirty microliters of cell suspensions (1 × 10^5^ cells/mL) were seeded into each channel of a microfluidic cell chamber, and 100 μL of the corresponding cell culture medium was added. The cells were cultured and stabilized in the incubator at 37 °C containing 5% CO_2_ and 95% air. The cells were incubated and cultured for 24 h under static conditions.

### 2.5. Cellular Uptake Using a Biomimetic Microfluidic System (BMS)

**Figure 2:**
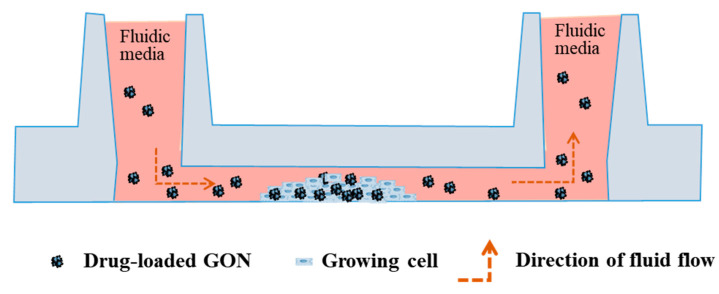
A cross-sectional view of a microfluidic cell chamber showing cellular behaviors of NPs under the biomimetic microfluidic system (BMS).

The microfluidic chamber was connected to the calibrated BMS, consisting of a peristaltic pump, media, microfluidic cell chamber, bubble trap, and tube, according to the previous studies [24,25]. The BMS, except the pump, was run in the incubator (37 °C and 5% CO_2_) during the whole experiment. The cell media containing GONs was delivered using the peristaltic pump throughout the microfluidic cell chamber, and the cell monolayer experienced shear stress, as shown in Figure 2. In this study, different levels of shear stress, i.e., 0.5, 5, and 50 dynes/cm^2^, were tested. For comparison, cells were also incubated with media containing GONs in the absence of microfluidic shear stress in static conditions. Cells were also incubated with only fresh cell media without adding GONs under the static condition as a control.

### 2.6. Determination of Cellular Uptake with C-GONs

#### 2.6.1. Flow Cytometry

The fluorescence intensity of coumarin-6 in C-GONs was quantitatively analyzed by the flow cytometer. After incubating cells with C-GONs for 1 h at 37 °C and 5% CO_2_ under static or dynamic conditions, the tubes were removed from the microfluidic cell chamber. The chambers were washed three times with phosphate-buffered saline (PBS, pH 7.0) for 5 min to discard the C-GONs from the cell suspension. Fifty microliters of a 0.25% (*w*/*v*) trypsin–EDTA solution were added to detach the cells for 3 min. Then, the cells were harvested by washing the channels twice with 100 μL of PBS. The mean fluorescence intensity of treated samples was analyzed using a flow cytometer (BD FACS Canto II, BD Biosciences, Franklin Lakes, NJ, USA). The geometric mean and median of 10,000 events for each sample were determined in triplicate. Control samples containing only cells without sample treatment were performed in triplicate.

#### 2.6.2. Confocal Laser Scanning Microscopy

The cells were incubated with C-GONs for 1 h at 37 °C and 5% CO_2_ under static or dynamic conditions. Then, the cells were washed thrice with PBS (100 μL, 5 min) to remove non-internalized C-GONs from the cell suspension. The cells were fixed with 4% paraformaldehyde in PBS (100 μL, 30 min) at 37 °C and washed twice with PBS for 5 min. The cell nuclei were stained with 3 μg/mL Hoechst #33342 (Invitrogen) in PBS for 15 min in the dark and washed twice with PBS. The images of the cells were visualized by fluorescence microscopy (Eclipse Ti-U, Nikon, Melville, NY, USA).

### 2.7. Cell Viability Assay with PTX-GONs

The cell viability assay was evaluated after exposing the cells to different levels of shear stress treated with PTX-GONs. The cells were seeded into a μ-slide chamber at a density of 3 × 10^5^ cells per well. Then, cells were incubated for 24 h. After growth to 90% confluence, the cells were washed twice with PBS to remove the residual growth medium. Cells were treated with 150 μL of fresh media that had PTX-GONs with different PTX concentrations (0.025, 0.125, 0.25, 2.5, 12.5, and 25 μg/mL) or blank GONs under static conditions for 24 h. The cells were also treated with 2.5 μg/mL PTX-GONs under fluidic shear stress conditions and incubated for 24 h. Cell viability was evaluated using the MTT assay. Briefly, cells were washed twice with PBS to eliminate the remaining drug, and 150 μL fresh medium was added, supplemented with 10% MTT solution and covered with tinfoil. After incubation at 37 °C for 1 h, the medium was withdrawn, and 150 μL of DMSO was added to dissolve the crystals. The resulting samples were gently shaken on an orbital shaker for 15 min. Finally, the supernatant was replaced on a 96-well plate. The absorbance of each well plate at 570 nm was determined using a Synergy H1 plate reader (BioTek, Winooski, VT, USA). These experiments were repeated independently, and the cell viability results were normalized to cells incubated in cell medium alone. The IC_50_ value was calculated by GraphPad Prism^®^ software (5.01, San Diego, CA, USA).

### 2.8. Statistical Analysis

The experimental data were shown as the mean ± standard deviation (SD). The statistical significance (*p*-value) of the data was analyzed by one-way analysis of variance (ANOVA) and a Tukey’s multiple comparison post hoc test was performed. A *p*-value was regarded as statistically significant at three levels: *, *p* < 0.05; **, *p* < 0.01; ***, *p* < 0.0001.

## 3. Results and Discussion

### 3.1. Physicochemical Properties: GONs, C-GONs, and PTX-GONs

The desolvation method was applied for the preparation of the GONs. Considering the solubility differences of the GOC in distilled water and ethanol, the desolvation method adequately induced the formation of a self-assembled GON system. The GOC was readily dissolved in 50% ethanol. The precipitation and nucleation of the GOC were controlled by the addition rate and volume of anhydrous ethanol for optimizing the characteristics of the GONs. The free amino groups of the GONs were then reacted with cross-linker glutaraldehyde for the further stabilization of the GONs. As summarized in Table 1, DLS results showed that the size of the GONs was around 200 nm, which could easily permeate into leaky tumor vasculature. The polydispersity index of the GONs was around 0.1, indicating a narrow particle size distribution of the NPs.

In contrast, the previously two-stage desolvation method for preparing the gelatin NPs showed a particle size of over 200 nm [26,27]. The cellular uptake of the 200–300 nm range of NPs was predominantly mediated by macropinocytosis [28]. In this study, DLS data exhibited different values of particle size in electron microscopes due to the hydration of soft particles and the different states of the sample. A low level of shear stress (0.5–15 dynes/cm^2^) or static conditions could induce the stable adhesion of NPs on cancer cells [29]. The adhesion of PTX-GONs to vasculature endothelial cells would be expected to be by enhanced permeation and retention (EPR) effects in terms of passive targeting. As they are regulated by the expression levels of adhesion molecules, the stages of adhesion of NPs could be classified by the uptake intensity of NPs [30].

**Figure 3:**
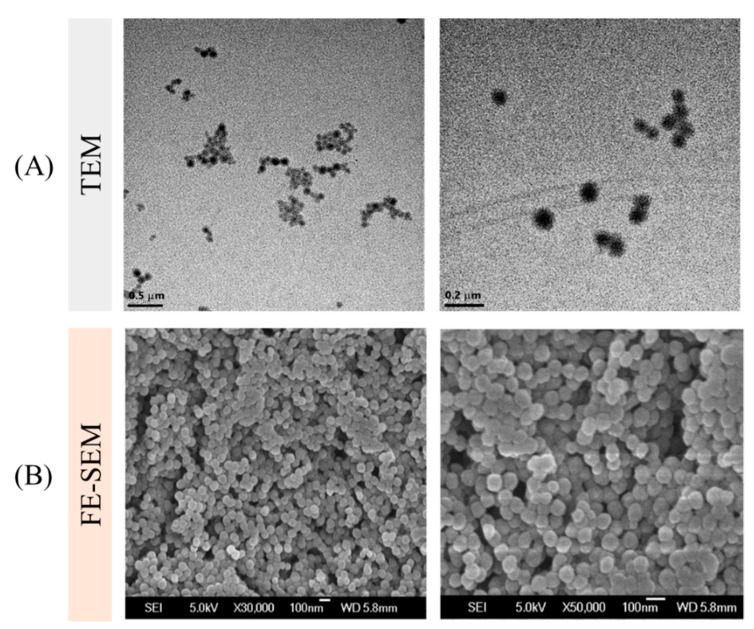
Electron microscopy images of GONs at two different magnifications (×30K and ×50K): (**A**) TEM and (**B**) FE-SEM.

In this study, a smaller particle size of GONs with high uniformity was produced by a one-step desolvation. Gelatin NPs have a low zeta potential of −10 mV [31]. The fattigation-platform GONs have a high absolute negative zeta potential, indicating the enhancement of colloidal stability [32]. It was expected that the oleic acid increased the hydrophobicity and improved the molecular interaction between the hydrophobic drugs and oleoyl carbon chain in the GONs. The amino groups of gelatin reacted with OA, which reduced the cationic charge on the GON surface. Accordingly, the surface charge of the GONs was electrostatically stabilized from the carboxyl groups (COOH) of gelatin with anionic surface charges. Figure 3 showed the electron microscope images of the GONs. These images supported the idea that GONs were spherical, compact, and uniform. Glutaraldehyde cross-linking agents permitted the GONs to be more stabilized via cross-linking and molecular compaction when dispersed in water. The cross-linked and stabilized GONs could provide more versatility in drug delivery for tumor targeting when the model drug is loaded.

**Figure 4:**
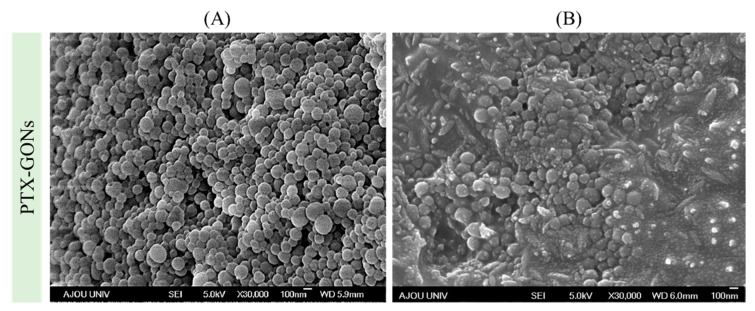
FE-SEM images of PTX-GONs according to states in the conditions: (**A**) the normal state and (**B**) the deformed state of PTX-GONs after shear stress was applied at 50 dynes/cm^2^.

To evaluate the cellular uptake of the GONs, coumarin-6 was encapsulated within the GONs (C-GONs) using the incubation process. The coumarin-6 was loaded into stabilized GONs through two potential mechanisms: (i) Penetrating into the pores of the GONs and (ii) absorbing through the outer layer of the GONs by physical absorption or a hydrogen bonding interaction. After the drug-loading process, the particle size of the drug-loaded GONs was increased. The absolute values of zeta potential were decreased. However, the zeta potential of the drug-loaded GONs was still negatively charged. It was assumed that the stability of the drug-loaded GONs was maintained in physiological conditions. PTX was successfully encapsulated into GONs with a DC of 8.15 ± 0.11% and an EE of 88.74 ± 11.62%. The previous study exhibited that the DC and EE of PTX-loaded D-α-Tocopherol polyethylene glycol 1000 succinate (TPGS) micelles were 3.09 ± 0.09% and 95.67 ± 2.84%, respectively [33]. Among the other PTX-loading vehicles, the PTX loading capacity of the GONs was quite comparable. Initially, the added amounts of PTX were set by considering the precipitation of the PTX-GONs. We tried the incubation drug loading process with 10 mg of GONs and different amounts at 2, 5, and 10 mg of PTX. However, the aggregation of the PTX-GONs quickly occurred over 2 mg of PTX. Figure 4 exhibited that the shapes of the PTX-GONs remained uniformly spherical after the highest degree of shear stress (50 dynes/cm^2^). These data indicated that the GONs were moderately deformed but not degraded and maintained their overall structure despite the dynamic conditions of the microfluidic system.

### 3.2. Cellular Uptake of Coumarin-6-Loaded GONs

It was known that the cellular behaviors of NPs are complicated by biomimetic shear stress depending on their physicochemical properties, such as components, charge, and biodegradability. It was known that poly(lactic-*co*-glycolic acid) NPs decreased targeting to epithelial or endothelial cells under shear stress [34]. Liposomes show different delivery efficiencies depending on their surface charges. Positively charged liposomes showed increased cellular uptake, whereas negatively charged liposomes showed decreased or no change in the cellular uptake of NPs in the presence of shear stress [12]. Additionally, negatively charged silica NPs showed increased cellular uptake at low shear stress. Previous studies showed that the intracellular uptake of positively charged cationic polystyrene NPs (PSNs) was highly increased in HEK 293T epithelial cell lines and MS1 endothelial cell lines under dynamic conditions compared to the static condition [25]. However, the negatively charged anionic PSNs showed no significant difference in cellular uptake with the presence of shear stress.

**Figure 5:**
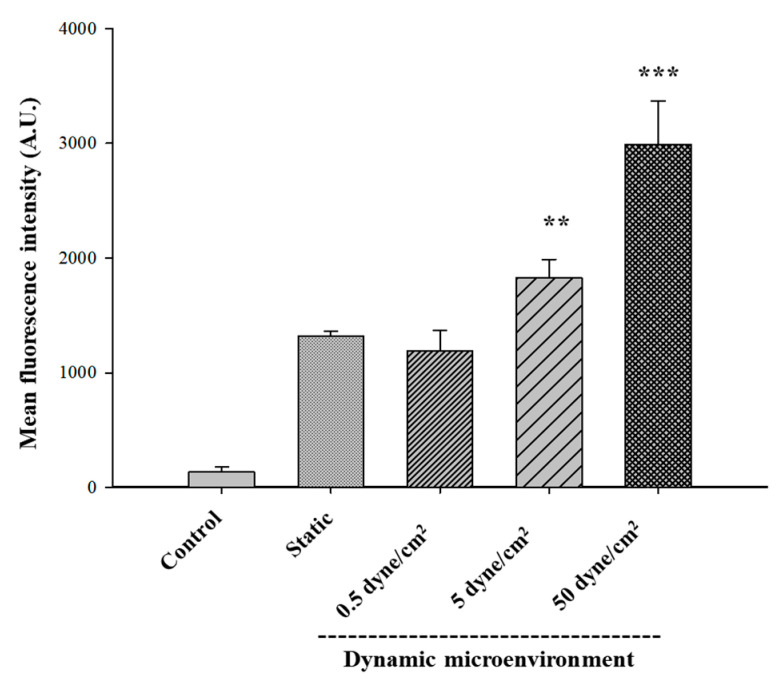
Cellular uptake of C-GONs by A549 cancer cells under a static or biomimetic dynamic microenvironment for 1 h (mean ± SD; *n* = 3, ** *p* < 0.01; *** *p* < 0.001).

**Figure 6:**
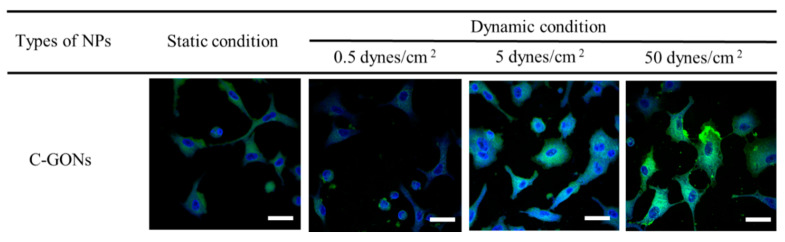
Confocal images of internalized GONs in A549 cancer cells after treatment with coumarin-6-loaded GONs under static or biomimetic dynamic conditions at different shear stress levels for 1 h (scale bar: 20 µm).

To compare the cellular internalization of GONs by cancer cells in the presence of shear stress, A549 cancer cell lines were selected for flow cytometry and confocal imaging. Several microfluidic system studies used the alveolar epithelial A549 cells as a model cell for explaining the intracellular mechanism and molecular signals coming from the fluidic shear force conditions in the vasculature [35]. Therefore, A549 cell lines were optimum model cells for designing the evidence-based microfluidic system. The cells were incubated for 1 h under static or biomimetic flow conditions with 0.5, 5, and 50 dynes/cm² shear stress. The quantitative evaluation by flow cytometry showed that the mean fluorescence of C-GONs was slightly decreased due to the presence of low shear stress, according to the increase in shear stress level (Figure 5). The cellular uptake of C-GONs at 5 and 50 dynes/cm^2^ was significantly higher compared to the static condition. The quantitative results from the flow cytometer corresponded to the qualitative results from the confocal laser scanning microscopy (CLSM) (Figure 6). The intensity of green fluorescence indicated the intracellular uptake of C-GONs, while the blue fluorescence image indicated the dyed cell nucleus. Confocal images clearly showed the increased green fluorescence of coumarin as the shear stress was increased as compared to the static condition cells. These results corresponded to previous research that negatively charged NPs had shown improved cellular uptake due to the presence of shear stress [25].

The cellular uptake studies of C-GONs confirmed that the cellular uptake of anionic C-GONs under dynamic conditions was significantly increased in comparison with the static condition, resulting in higher cell-killing efficiency under dynamic conditions. It was proven that the cationic NPs showed shear stress-dependent cellular uptake, while the anionic NPs were less affected by shear stress in the cellular microenvironment. Our results supported the idea that the surface charge of NPs was a critical parameter of cellular uptake. Strong negatively charged particles required more time to be taken by cells via endocytosis or active transport due to the strong repulsion between negatively charged groups and the surface of the cell membrane [36]. However, the interaction between particles and cells was somewhat constrained by the fluid shear force at lower levels.

Additionally, NPs were exposed to extracellular proteins in vivo environment and adsorbed proteins on their surface, forming the protein corona [37]. The albumin protein corona assisted the specific cellular receptors in mediating the intracellular transport pathway of fattigation-platform NPs [38]. The surface charge of NPs was a crucial factor in determining the cellular uptake of the NPs that could bind to the surface of the cell membrane and define the possible uptake routes of the intracellular pathway [39]. In the case of albumin NPs, the protein layer of albumin had a conflicting effect. The cellular binding of protein–cationic NP complexes was improved. However, the cellular binding of protein–anionic NP complexes was inhibited due to the different cellular receptors [40]. We hypothesized that the protein corona on the surface of NPs might be detached under the increased force of shear stress at 50 dynes/cm^2^, which might allow NPs to diffuse more into the cancer cells either by the enhanced permeability and retention effect or via increased interaction with intracellular cell adhesion molecules [41]. Consequently, flow shear stress stimulated cellular behaviors and the uptake of GONs interacting with the cell adhesion molecules of A549 cancer cells.

### 3.3. Cell-Killing Efficiency of PTX-GONs

**Figure 7:**
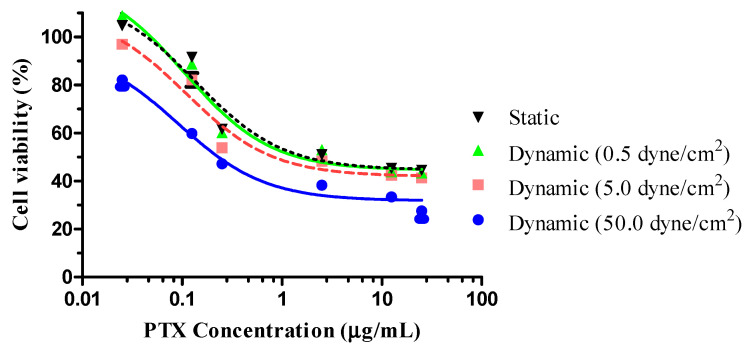
Cell viability of A549 cells treated with PTX-GONs under different cell culture conditions incubated for 24 h (data are expressed as mean ± SD for triplicate experiments, *n* = 6).

The cell-killing efficiency of PTX-GONs in static and dynamic conditions was evaluated by an MTT assay (Figure 7). The IC_50_ value of PTX corresponded to the plasma levels of the drug achievable in human subjects. The decrease in cell viability, as measured by the MTT test, may have resulted in the inhibition of cell growth or cytotoxicity. The cells were also incubated with drug-free GONs to ensure that the cytotoxicity was caused by PTX. The vehicle of NPs used to test cytotoxic activity had the same concentration of blank GONs as the sample containing the highest concentration of PTX (25 μg/mL). No cytotoxicity activity was observed for the blank GONs, even in the presence of shear stress. Therefore, the cytotoxicity of PTX-GONs was attributed only to PTX, rather than GONs consisting of non-toxic and biocompatible materials. These results suggested that GONs maintain the pharmacological activity of PTX and efficiently deliver the PTX to the cells. The cytotoxicity of the PTX-GONs was dependent on the dose concentration of PTX. An MTT assay was performed on PTX-GONs under static and dynamic conditions to compare the effects of fluidic shear stress on drug efficacy.

As summarized in Table 2, The IC_50_ of the PTX-GONs was found to be dose-dependent and shear stress-dependent. The IC_50_ of PTX-GONs under static conditions was 0.1377 ± 0.0622 µg/mL. Referring to the cytotoxicity results from the literature with the PTX-loaded drug delivery system, the IC_50_ value of free PTX on A549 cells was 5.21 ± 0.93 µg/mL after 24 h treatment [33]. Based on the IC_50_ value in a static condition, the PTX-GONs showed a 37.83-fold higher cytotoxic effect as compared to the free PTX. In the case of the dynamic conditions, the IC_50_ values of the PTX-GONs for 0.5, 5, and 50 dynes/cm^2^ were 0.105 µg/mL, 0.108 µg/mL, and 0.091 µg/mL, respectively. There was a significant difference in the IC_50_ values of the PTX-GONs at 5 and 50 dynes/cm^2^ as compared to the control groups. The cellular cytotoxic effects of the PTX-GONs were moderately higher at 50 dyne/cm^2^ compared to 0.5 and 5 dyne/cm^2^ in dynamic conditions, as well as in the static condition, which indicated the superior cytotoxicity.

These outstanding results demonstrated the roles of diverse shear stress microenvironments observed with the uptake of the drug delivery vehicle as compared to the system in which the efficacy of a drug delivery vehicle was only evaluated at a single shear rate. The interaction of the NPs with cells depends on the specific properties of the nanoparticles, including material and size, as well as the surface charge of the NPs. Considering the particle size of the NPs, the achieved results showed that after incubation with coumarin-6 or PTX, the particle size of the GONs increased as compared to the initial GONs, especially PTX-GONs with nearly double particle size. This phenomenon could be attributed to the adsorption of a chemical agent onto the surface of the NPs. The adsorption of PTX, whose molecular structure was more complicated than coumarin-6, led to a more significant change, resulting in the particle size of the GONs.

Unlike the cellular behaviors of PSNs or liposome (Doxil^®^) previously investigated by our research groups [24], the cell viability of the PTX-GONs under dynamic conditions at 0.5 dynes/cm^2^ was slightly higher than those of the PTX-GONs in a static condition. As shown in Figure 5 and Figure 6, these findings showed the identical cellular uptake of the C-GONs obtained in the presence of shear stress. Although the PTX-GONs possessed a larger particle size than the C-GONs, those two types of drug-loaded GONs are still negatively charged and display a similar tendency in cellular uptake. Interestingly, previous studies reported that a high level of shear stress triggered the apoptosis of tumor cells and the death of circulating tumor cells (8–60.5 dynes/cm^2^ in HCT116 and 60 dynes/cm^2^ in engineered MDA-MB-231 circulating tumor cells) [42,43]. This research indicated that the intensities and incubation time of shear stress was functionalized in critical parameters for the cellular behaviors of NPs. Our BMS stably held the cells on during the dynamic culture conditions. High shear stress increased the exposure time of the soft GONs in the experimental design. Ultimately, the soft GONs could interact with adhesion molecules on A549 cell lines. Therefore, it is suggested that the shear stress seemed to play a more crucial role, along with particle size, in the cellular internalization process.

The materials of the NPs were the critical factor for determining the efficacy and toxicity in drug delivery because the particle elasticity and deformability of NPs could modify cellular penetration, blood circulation, tissue targeting, and specific interactions with cells [44]. For example, softer NPs exhibited significantly reduced cellular uptake in in vitro studies and enhanced circulation and subsequently enhanced targeting as compared to harder NPs in vivo [45]. However, in the present study, the GONs, which were negatively charged and soft-structured NPs, were less affected by the dynamic environment at the higher rate of shear stress and maintained their overall structure, which might be due to cross-linking by EDC–NHS, as well as glutaraldehyde. It was reported that the conformation of the NPs could be controlled by their intrinsic rigidity and coronal interaction with the fluidic substrates [46]. It was assumed that the covalent conjugation of gelatin and oleic acid, as well as cross-linking by glutaraldehyde for structural reinforcement, could impart the overall gel-like rigidity to the NPs, and thus prevent deformation under high shear stress conditions. However, the detailed elasticity and cellular uptake of the GONs under the high shear stress needs to be investigated. Under the biomimetic fluidic conditions, cancer cells were exposed to diverse physical forces in the tumor microenvironment, such as fluidic shear stress, hydrostatic pressure, tension, and compression [47]. Shear stress was generated by fluidic flow encountering the surface of the cell monolayer, which could promote mass transport and modulate cellular dynamic activities; thus, shear stress could affect the cell viability and metastasis of cancer [48].

Additionally, it was desirable to investigate in more detail the interactions between NPs and cells having different physicochemical properties and protein corona under a biomimetic dynamic environment simulating in vivo. Our research could provide a way to bridge the in vitro–in vivo gap with 3D models or co-cultures, combined with the current dynamic fluidic system. Utilizing a simple BMS could allow a better predictive understanding of in vitro cellular behaviors and drug distribution in tumor targeting under in vivo environments. Consequently, these results further emphasize the need for the evaluation of cellular interactions with nanoparticle drug delivery systems according to their physicochemical properties, such as type of material, size, and surface charge under biomimetic and disease-specific microenvironment conditions.

## 4. Conclusions

We investigated the effects of variable fluidic shear stress on drug delivery using well-established fattigation-platform GONs. The cellular uptake of C-GONs, as a function of biomimetic shear stress, revealed significantly improved uptake intensity compared to the static condition and the dynamic condition with different levels of shear stress. PTX-GONs showed enhanced cancer cell killing effects and a lower IC_50_ under dynamic conditions at higher shear stress (50 dynes/cm^2^) compared to static conditions and lower shear stress (0.5 and 5 dynes/cm^2^). We could assume that higher shear stress might lead to an increase in the diffusion of soft and deformable GONs to the cancer cells and induce the apoptosis of the cells, resulting in enhanced cell-killing efficiency.

We established our BMS device that permitted the culturing of epithelial cells under different levels of shear stress in the dynamic conditions that simulate the cancer microenvironment in vivo and also suggested the cellular behaviors of NPs between the shear stress and the EPR effect. The in vitro microfluidics platform device has been faced with several technical challenges with failure to imperfectly reproduce the parameters or values of hemodynamics occurring in vivo. Recently, with the application of microfluidic studies on cancer, metastatic patterns, and the microenvironment of cancer have been scrutinized [35,49]. This research area will evolve to reduce unnecessary in vivo experiments and to accelerate the construction of artificial vascular and multi-organ systems for avoiding the invasive monitoring of the body. Therefore, the evolution of this research field, various stages of cancer microenvironments, and the impact of shear stress could provide milestones for consequent studies on microfluidics.

## Figures and Tables

**Table 1:** Physicochemical properties of gelatin–oleic NPs (GONs), coumarin-6-loaded GONs (C-GONs), and paclitaxel-loaded GONs (PTX-GONs). Data are expressed as mean ± SD, *n* = 3.

Type of NPs	Particle Size (nm)	PDI *	Zeta Potential (mV)
GONs	163.23 ± 7.91	0.105 ± 0.03	−64.62 ± 1.37
C-GONs	199.07 ± 1.48	0.164 ± 0.03	−36.11 ± 8.28
PTX-GONs	309.21 ± 3.56	0.064 ± 0.05	−23.73 ± 1.18
PTX-GONs after 50 dynes/cm^2^	291.48 ± 4.72	0.512 ± 0.08	−21.44 ± 3.51

* PDI: polydispersity index.

**Table 2:** IC_50_ values (µg/mL) of PTX-GONs at different cell culture conditions (data are expressed as mean ± SD, *n* = 6).

Cell Culture Conditions	IC_50_ Values (µg/mL) of PTX-GONs
Static condition	0.1377 ± 0.0622
Dynamic condition at 0.5 dynes/cm^2^	0.1057 ± 0.0474
Dynamic condition at 5 dynes/cm^2^	0.1084 ± 0.0584
Dynamic condition at 50 dynes/cm^2^	0.0914 ± 0.0465

## Abbreviations

NPs	Nanoparticles
GOC	Gelatin–oleic Conjugate
GON	Gelatin–oleic Nanoparticles
PTX-GONs	Paclitaxel-Loaded GONs
C-GONs	Coumarin-6-Loaded GONs
TEM	Transmission Electron Microscopy
SEM	Scanning Electron Microscopy
DC	Drug Loading Content
EE	Encapsulation Efficiency
BMS	Biomimetic Microfluidic System
IC_50_	Inhibitory Concentration
